# An Energy Dissipative Binder for Self‐Tuning Silicon Anodes in Lithium‐Ion Batteries

**DOI:** 10.1002/advs.202205443

**Published:** 2022-11-17

**Authors:** Yihong Tong, Siyu Jin, Hongyuan Xu, Jiawei Li, Zhao Kong, Hong Jin, Hui Xu

**Affiliations:** ^1^ Suzhou Academy Xi'an Jiaotong University Suzhou 215123 China; ^2^ Nano Science and Technology Institute University of Science and Technology of China Suzhou 215123 China; ^3^ Suzhou Institute for Advanced Research University of Science and Technology of China Suzhou 215123 China; ^4^ Sustainable Energy Laboratory Faculty of Materials Science and Chemistry China University of Geosciences Wuhan 430074 China

**Keywords:** binder, citric acid, energy dissipation, lithium‐ion batteries, silicon anode

## Abstract

The volume change of the silicon anode seriously affects the electrode integrity and cycle stability. Herein, a binder, GCA13, with energy dissipation function and surface stability effect is proposed to enhance the cycle life and specific capacity. Unlike traditional binders that protect silicon electrodes through long‐chain networks, GCA13 introduces citric acid molecules with short‐range functions on the long‐chain guar gum through weak interconnection. This short‐range action is similar to the function of a spring, which can effectively buffer the silicon particle pulverization caused by the volume change. Therefore, the electrode can effectively maintain structural integration with ignorable cracks and alleviated thickness swelling. Thus, the Si@GCA13 anode exhibits a high reversible capacity of 1184 mAh g^−1^ under 2 A g^−1^ after 740 cycles with a latter coulombic efficiency of 99.9%. Extraordinarily, benefiting from the superior properties of the GCA13 binder, the electrode shows remarkable cycling stability under low (−15 and 0 °C) and high temperatures (60 °C). The work demonstrates the great potential of this binder design strategy to achieve the overall property promotion of Si anodes for practical application even under harsh service conditions.

## Graphical abstract

An energy dissipative binder is designed to achieve stable cycling of Si anodes within −15–60°C. By plasticization of small molecules, this viscoelastic binder enables the electrode remarkable self‐tuning and self‐healing abilities via particle rearrangement. This binder also induces a stable bilayer solid electrolyte interphase to ensure outstanding long‐term performance of the Si anode.

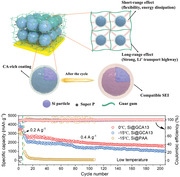

## 1 Introduction

Silicon is one of the most promising anode materials due to its high specific capacity (3590 mAh g^−1^ for Li_3.75_Si at room temperature and 4200 mAh g^−1^ for Li_4.4_Si at 415 °C), low operating voltages (≈0.5 V vs Li/Li^+^), and vast reserves.^[^ ^1^ ^]^ Nevertheless, practical applications of silicon anodes are limited due to repeated huge volume changes (as much as 300%) that result in pulverization of Si particles,^[^ ^2^ ^]^ loss of electrical contact,^[^ ^3^ ^]^ and incompatible solid electrolyte interphase (SEI),^[^ ^4^ ^]^ and therefore, rapid capacity decay.^[^ ^5^ ^]^ Apart from designing nanostructures or composites, researchers endorse that the development of Si anodes is a systematical work.^[^ ^6^ ^]^ Only if the appropriate binders and electrolytes are developed, the advantages of Si anodes could be fully exploited to achieve overall good performance.^[^ ^7^ ^]^ Thus, though a small content of the binder is utilized in the electrode, a remarkable effect can be made in terms of the electrode stability and cycling life.^[^ ^8^ ^]^

The mechanical property of the binder is crucial to determine the cycling stabilization of Si anodes.^[^ ^9^ ^]^ It highly depends on the polymeric molecular configuration, as well as intramolecular and intermolecular interactions within the binder network.^[^ ^10^ ^]^ The adjustment of the molecular network can be achieved by routine approaches, such as mixing,^[^ ^11^ ^]^ grafting,^[^ ^12^ ^]^ and cross‐linking,^[^ ^13^ ^]^ of polymeric components to improve the strength or toughness of the binders.^[^ ^9^ ^]^ Generally speaking, rigid networks formed by hard polymer backbones or covalently cross‐linked configuration exhibit high strength but insufficient toughness to withstand structural damage.^[^ ^14^ ^]^ On the other hand, highly elastic networks constructed by soft chains or flexible interactions could adapt to large volume changes of Si particles, however, may lack ductility to dissipate energy.^[^ ^15^ ^]^ Other than these two conventional approaches, a novel strategy for binder designing to build a pliable and tough network to maintain electrode structural integrity is highly desirable.^[^ ^5^, ^15^ ^]^ Therefore, the relevant mechanisms and methods for an optimized binder to achieve all‐around properties promotion of Si anodes are keen for a thorough investigation.^[^ ^16^ ^]^

Under proper designing, the binder can induce the beneficial self‐healing property to Si anodes generally by introducing reversible chemical bonds such as borate ester bonds,^[^ ^17^ ^]^ hydrogen bonds,^[^ ^18^ ^]^ ionic dipole interactions,^[^ ^19^ ^]^ supramolecular interactions,^[^ ^20^ ^]^ and dynamic covalent interactions.^[^ ^5^, ^15^ ^]^ However, due to the diluted active sites resulting from the low content of the binder in the electrode, the crack‐recovery phenomenon is much harder to observe on the cycling electrode than on the pure polymer samples according to most published papers to date.^[^ ^18^, ^21^ ^]^ Therefore, an advanced self‐healing strategy for long‐cycling electrodes based on the optimized binder properties is worth further digging out. In short, an ideal binder is expected to robustly support the electrode structure via its superb mechanical network.^[^ ^22^ ^]^ If it can also induce self‐healing ability and other multiple functions, such as ionic‐conductive^[^ ^23^ ^]^ or interface stabilizing^[^ ^24^ ^]^ effect, it will be extensively favorable to further enhance the overall performance of silicon anodes.

**Scheme 1:**
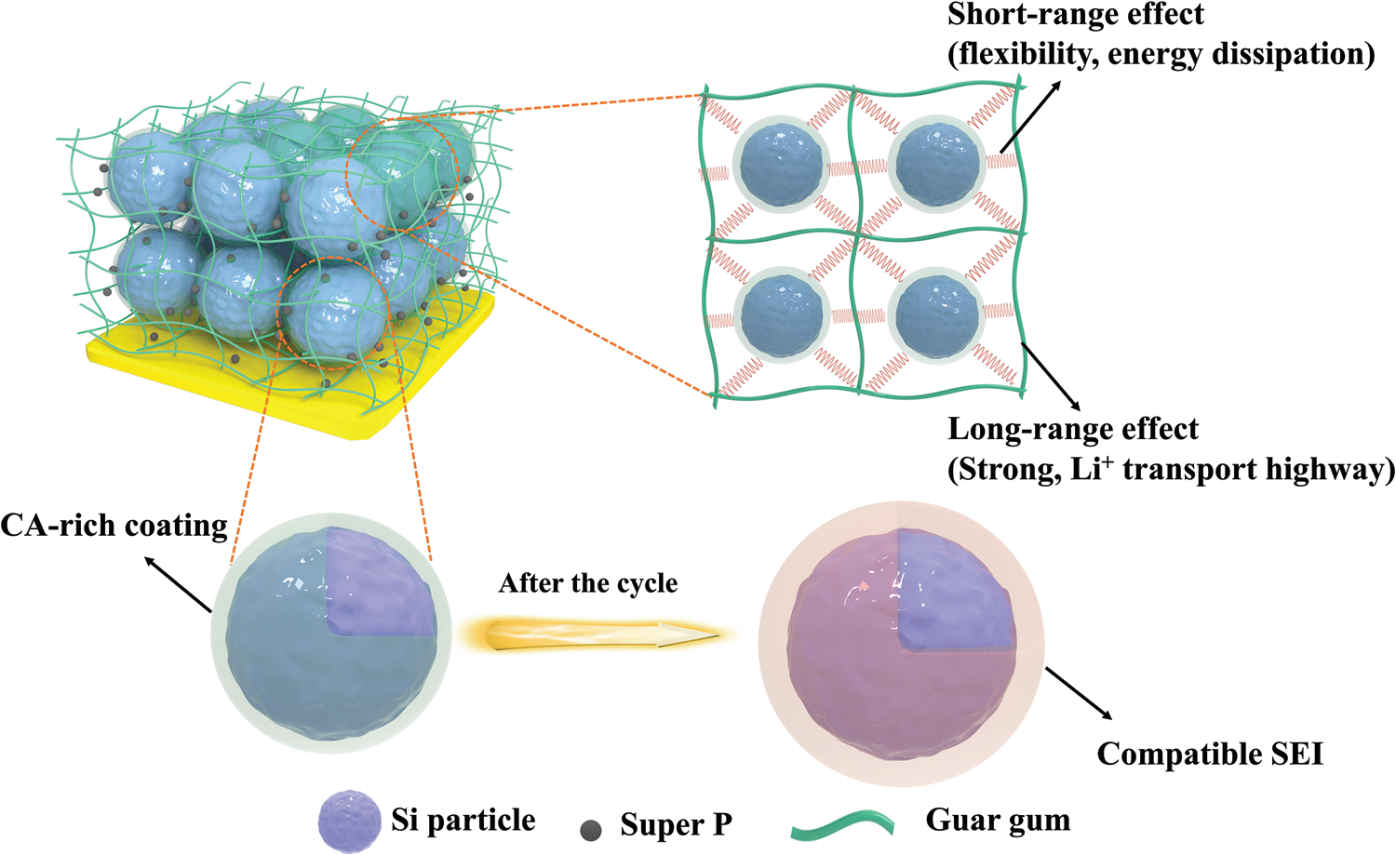
Proposed work mechanism of the multifunctional binder (GCA13) in Si anode.

In order to address the aforementioned issues, herein, we designed a multifunctional binder (termed GCA13) to realize comprehensive promotion of properties of silicon anodes. As shown in **Scheme** **1**, this binder is co‐engineered by the long‐range effect formed by the guar gum (GG) polymeric backbones and the short‐range effect induced by citric acid (CA) small molecules. Due to the plastification of the CA molecules, the GCA13 binder displays a unique viscoelastic property. This ductile and tough binder network gives rise to the Si anodes a self‐tuning ability, enabling Si particles free to rearrange when encountering volume change. From this, the integration of the electrode effectively maintains via self‐healing cracks and reorganizing structure during cycling. Therefore, to synergetically combine the strong long‐range support of binder and flexible short‐range effect induced by the self‐tuning process, the electrode can remain structurally stable upon Si volume change. Besides, the advantageous mechanical support through the above mechanisms, this binder also has a significant stabilizing effect on the electrode interface. By inducing the formation of a pliable double‐layer SEI, the electrode interface shows an enhanced energy dissipated ability and noticeable electrochemical stability. Benefited from this multifunctional binder, the Si anode shows outstanding performance with a capacity of 1184 mAh g^−1^ after 740 cycles at 2 A g^−1^, and an extended application under a wide temperature arrange (at −15, 0, and 60 °C). The investigation of the self‐tuning mechanism demonstrates the promising perspective of this binder‐designing strategy based on appropriate molecular adjustment.

## 2 Results and Discussion

In order to achieve stable cycling of Si anodes in a wide temperature range, a multifunctional binder GCA13 has been developed to induce self‐tuning and self‐healing properties for the Si electrode. By combining GG chains as the backbones and CA small molecules as the grafting, a long‐range effect (to provide strong mechanical support and Li^+^ transport highway) and a short‐range effect (to provide structural flexibility and energy dissipation upon Si volume change) was synergistically designed to construct a robust network, as shown in Scheme 1. The free CA molecules can plastify the binder network and enhance its viscoelasticity according to the free volume theory. It gives rise to the electrode a structural self‐tuning ability via particle rearrangement of the Si particles upon huge volume variation during cycling to release stress and alleviate electrode swelling. Synergistic affecting with the abundant reversible hydrogen bonds in the binder network, the Si anodes show an outstanding self‐healing ability to recover cracks and damages. In addition, the binder induces a CA‐rich coating on the silicon surface that can help maintain the interface stability by inducing a LiF‐rich double‐layer SEI with appropriate modulus and protection from the continuous decomposition of the electrolyte. Resulting from the superior mechanical support and interface stabilizing effects from this multi‐functional GCA13 binder, this unique design strategy on the viscoelastic network enables an excellent electrochemical performance of the Si anode especially on long cycling, as well as low and high‐temperature conditions.

**Figure 1:**
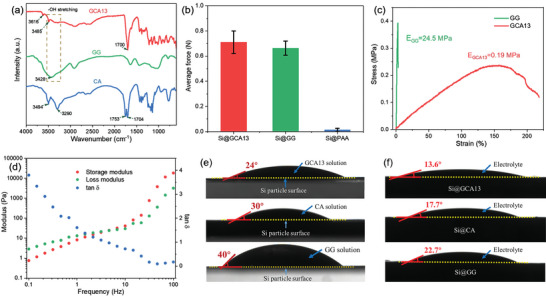
a) FTIR spectra of GCA13, GG, and CA. b) Average adhesion force of Si electrode using different binders. c) Stress–strain curve of GCA13 and GG polymer films. d) Frequency‐dependent viscoelastic modulus test of GCA13 solution (1wt%). e) Digital photographs of the contact angle of 1wt% GCA13, CA, and GG solution with SiNPs. f) Digital photographs for electrolyte (1 m LiPF_6_ in EC:DEC = 1:1, add 10% FEC and 1% VC) contact angle of SiNPs composited with GCA13, CA, and GG binders.

The as‐synthesized GCA13 binder was verified by X‐ray photoelectron spectroscopy (XPS) and Fourier transform infrared (FTIR) spectra. Strong and sharp 1s peaks at 532.5 and 284 eV in the XPS spectra of GCA13 and GG confirm the obvious existence of C and O elements (Figure S3a, Supporting Information). As shown in Figure S3b, Supporting Information, the high‐resolution C1s spectra of GCA13 can be resolved into three peaks corresponding to O=C—O at 289.2 eV, C—O at 286.7 eV, and C—C at 284.8 eV, showing ether bonds, hydroxyl, and carboxyl groups in GCA13. In addition, the high‐resolution O1s spectra of GCA13 display two peaks of C=O at 532.1 eV and C—O at 533.1 eV (Figure S3c, Supporting Information).^[^ ^25^ ^]^ **Figure** **1**a shows the FTIR spectra of CA, GG, and GCA13. GG displays a broad peak at 3428 cm^−1^ described as the stretching vibration of O—H. For CA, the broad peaks at 3496 and 3290 cm^−1^ correspond to the O—H stretching vibrations of —COOH, while the double peaks at 1753 and 1704 cm^−1^ correspond to the symmetric and asymmetric vibrations of C=O, respectively.^[^ ^26^ ^]^ Compared with CA, GCA13 appeared at a new peak at 3616 cm^−1^ corresponding to free C=O, indicating the existence of free carboxyl groups. The C=O double peaks in citric acid turn into a single peak and shift to a low wavenumber (1700 cm^−1^), and the O—H stretching vibration in COOH shifts to a low wavenumber (3485 cm^−1^), indicating the formation of a hydrogen bond.^[^ ^27^ ^]^ Meanwhile, it is beneficial to improve binder performance by generating strong interfacial interaction with the silicon surface. The abundant —OH and —COOH on GCA13 can form strong interactions with the silicon dioxide layer on the SiNPs surface through hydrogen bonding and ester bonding, which were confirmed by FTIR spectroscopy (Figure S4, Supporting Information). As shown in Figure S4, Supporting Information, after mixing with SiNPs, the peaks of 3485 and (corresponding to —OH stretching vibration in COOH) 3616 cm^−1^ (corresponding to free C=O) in GCA13 disappeared and the absorption peak of C=O shifted (1735 cm^−1^), which is due to the formation of hydrogen and ester bonds that change the electron cloud density.^[^ ^26^, ^28^ ^]^ To further determine the role of CA, transmission electron microscopy (TEM) tests were performed on the as‐prepared electrode materials. Elemental mapping images (Figure S5, Supporting Information) exhibit uniform distribution of C, O, and Si elements, confirming that the Si particles were well coated by a CA‐rich layer. From the TEM images shown in Figure S6, Supporting Information, the thickness of the coating was estimated to be around 5 nm. This coating is the foundation of inducing the local short‐range effect to stabilize the electrode, as well as forming the compatible LiF‐rich SEI to stabilize the particle interface during cycling.

To verify the wetting properties of the binder, the contact angle test was performed and displayed in Figure 1e,f. The results show that, for the surface of SiNPs, the GCA13 binder solution delivers a contact angle of 24°, which is smaller than that of the citric acid solution (30°) and the guar gum solution (40°). This illustrates an excellent wettability of GCA13 binder on the Si surface, which can lead to a uniform spreadability of binder in the electrode. Regarding the wettability of the electrolyte on various as‐prepared electrodes, Figure 1f shows that Si@GCA13 has the smallest contact angle (13.6°) compared with Si@CA (17.7°) and Si@GG (22.7°), suggesting that the Si anodes made by GCA13 binder can be efficiently activated during cycling.

An ideal binder should have strong adhesion both to the current collector and the electrode materials.^[^ ^6c^ ^]^ Adhesion test and 180° peeling test were applied to evaluate the adhesive properties of the binder. As seen from Figure 1b; Figures S7 and S8, Supporting Information, GCA13 polymer film has strong adhesion to an 8 g full bottle without dropping down. The peeling test displays that the Si@GCA13 electrode shows a high adhesion strength of 0.71 N to the copper foil, while that of Si@GG and Si@PAA electrodes is only 0.66 and 0.02 N, respectively. Furthermore, GCA13 polymer films present outstanding mechanical properties. The tensile test exhibits that the GG polymer film showed a poor maximum strain (3%) with an elastic modulus as high as 24.5 MPa, suggesting a relatively stiff property. In contrast, the GCA13 film displayed a much higher maximum strain (over 160%) with an elastic modulus of 0.19 MPa (Figure 1c and Figure S9, Supporting Information), implying a better elasticity of GCA13 than the pure GG binder. Moreover, rheological tests were performed to verify the viscoelasticity of the GCA13 binder. The curves in Figure 1d clearly show the coexistence of storage modulus (*G*′) and loss modulus (*G*′′) at all frequencies, indicating that GCA13 binder exhibits both elastic and viscous properties. This unique viscoelasticity of the GCA13 binder probably results from the plastification of CA molecules according to the free volume theory. The synergistic reaction of the long‐range effect (in terms of the binder backbones) and the short‐range effect (in terms of the local interactions with Si particles) can significantly benefit the overall properties of the Si anode. When suffering from the huge Si volume change, it is expected that the viscoelastic binder network could help maintain the integration of the electrode by recovery and rearrangement of the Si particles. Thereout, this may bring about self‐tuning and self‐healing abilities, which can remarkably promote the electrode robustness and elevate the electrochemical performance of the Si anode during cycling.

**Figure 2:**
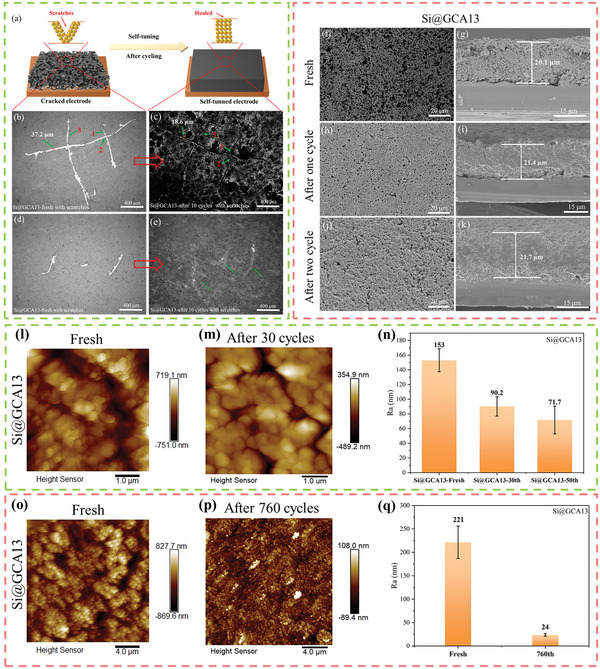
a) Schematic diagram of self‐tuning. Top‐viewed OM images of Si@GCA13 electrode with scratches, b,d) fresh, c,e) after ten cycles (the bright part in the figure is the exposed copper foil). Top‐viewed SEM images of Si@GCA13 electrode, f) fresh, h) after the first cycle, j) after two cycles. Cross‐sectional SEM images and thickness of Si@GCA13 electrode, g) fresh, i) after one cycle, k) after two cycles. AFM images of Si@GCA13 electrode, l,o) fresh, m) after 30 cycles, p) after 760 cycles. n,q) Comparison of the mean roughness (*R*_a_) of Si@GCA13 electrode at different cycles.

The self‐healing property of binders can facilitate crack repair, which plays an important role in maintaining the structural integrity of the electrode.^[^ ^29^ ^]^ The self‐healing performance of the GCA13 binder was evaluated by observing the morphological evolution both on a dissected polymer film and the scratched electrode during cycling (will be discussed later in **Figure** **2**). In Figure S10, Supporting Information, when the two completely severed parts of polymeric GCA13 film were in contact, the two pieces joined and healed spontaneously, without breaking apart even hanging onto a 5.2 g glass slide (much heavier than its own weight). This obviously indicates the excellent self‐healing ability of the GCA13 binder. In strong contrast, the GG polymer film cannot be detected a noticeable self‐healing phenomenon (Figure S11, Supporting Information). Though GG contains a great amount of hydroxyl groups, its stiffness impedes the formation of a strong reversible network. However, the addition of abundant CA molecules significantly increased the active sites to construct multiple hydrogen bonds between the binder backbones (Figure S12, Supporting Information). Moreover, the presence of CA largely modifies the flexibility of the binder network to further enhance the recovery capability of the bonds upon breaking.

**Figure 3:**
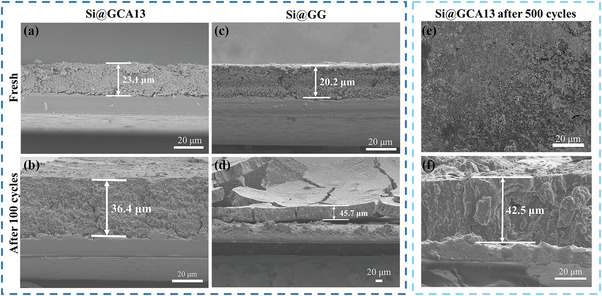
Cross‐sectional SEM images and thickness of Si@GCA13 electrode, a) fresh, b) after 100 cycles. Cross‐sectional SEM images and thickness of Si@GG electrode, c) fresh, d) after 100 cycles. e) Top‐viewed SEM images and f) cross‐sectional SEM images of Si@GCA13 electrode after 500 cycles.

To confirm the self‐healing performance of silicon anodes prepared by GCA13 binder, the morphology evolution of the scratched electrode was investigated under optical microscopy (OM) and scanning electron microscopy (SEM). As shown in Figure 2, Figure 2b is a fresh silicon anode with preformed scratches of about 0.8–2.3 mm in length (the bright part is the exposed copper foil). In Figure 2c, after ten cycles, the scratches at marked positions 1 and 2 showed significant healing. The groove width at position 3 decreased from 37.2 to 18.6 µm. This remarkable self‐healing phenomenon of Si@GCA13 electrodes is thought mainly attributed to the rearrangement of the Si particles during cycling as shown in Figure 2a. To verify this self‐healing mechanism, the top‐viewed and cross‐sectional morphologies before and after cycling were compared for Si@GCA13 anodes. From Figure 2f–k, the porous electrode (due to the freeze‐drying approach) gradually becomes denser with a slight thickness increase as the cycling progresses (20.1 to 21.4 to 21.7 µm, from fresh to the first to the second cycles). Till the 100th cycle (Figure S13, Supporting Information) and the 500th cycle (**Figure** **3**e,f), the structure turns out to be very dense and uniform without noticeable cracks, indicating an apparent particle rearrangement and self‐tuning process in the electrode. This self‐healing and self‐tuning ability of the electrode is beneficial from the viscoelastic properties of the GCA13 binder, which originates from the plastification of CA molecules. Besides, the synergistic effects of long‐range functions from the backbones and local short‐range functions from small molecules further help maintain the integration of the Si anodes. In addition, the porous structure formed by the freeze‐drying method not only provides reserve voids to accommodate the volume expansion of silicon but can also leave sufficient room for particle rearrangement during cycling.

The atomic force microscopy (AFM) test was also used to further verify the structural rearrangement of the electrodes. Figure 2l,m,o,p exhibits the 3D surface topography images of the Si@GCA13 electrodes before and after cycling. The surface height variation of the electrode gradually decreases during cycling. For one sample, it ranges from ≈−751–719.1 nm for fresh electrodes and then decreases to ≈−489.2–354.9 nm after 30 cycles. For another sample, the height variation ranges from ≈−869.6–827.7 nm for fresh electrodes and reduces to ≈−89.4–108 nm after 760 cycles. For the average roughness *R*_a_ of the electrodes in Figure 2n,q, it drops from 153 to 90.2 nm and 71.7 nm for the electrode from fresh to 30‐ and 50‐cycle, respectively. For the other sample, after 760 cycles, the *R*_a_ of the fresh electrode changed greatly from 221 to 24 nm. The surface smoothing of the electrodes further confirms the Si particle rearrangement under the condition of the GCA13 binder.

In order to study the enhancement of the GCA13 binder on the structural stability of silicon anodes, the morphology and thickness of the electrodes before and after cycling were analyzed by SEM. Figure S13a–f, Supporting Information, shows that, after 100 cycles, the surface of the Si@GCA13 electrode was smooth without noticeable cracks. In contrast, there are several large cracks can be seen in the cycled Si@GG and Si@CA electrodes. Moreover, it can be seen from Figure 3 that the thickness swelling of the Si@GCA13 electrode is about 158% (from 23.1 to 36.4 µm) after 100 cycles, which is much smaller than that of the Si@GG electrode (226%, from 20.2 to 45.7 µm). In particular, the Si@GG electrode exhibited a severe fracture and exfoliation after 100 cycles (Figure 3d), while the Si@GCA13 electrode still maintained an integrated structure with only 184% thickness swelling even after 500 cycles (Figure 3e,f). These results clearly indicate that the GCA13 binder can effectively alleviate the influence of Si volume change through the self‐tuning and self‐healing effects to achieve the structural integrity of the electrode during long cycling.

An electrochemically stable interface is essential to the stable cycling performance of the electrode. To confirm the impact of the binder on the interface, the chemical composition of the SEI (in the surface layer of the silicon electrode) after various cycles was analyzed. Figure S15, Supporting Information, depicts the XPS spectra of the Si@GCA13 electrode at different cycles. As can be seen from Figure S15a–c, Supporting Information, for the fresh electrode, the C1s spectrum exhibits peaks at 284.8, 286.6, and 288.6 eV corresponding to C—C/C—H, C—O, and C=O bonds, respectively. The peaks at 531.7 and 532.9 eV in the O1s spectrum correspond to C=O and O—C=O signals, respectively. After five cycles, the peak of F1s appeared (Figure S15c, Supporting Information), which was due to the decomposition of electrolytes during the formation of SEI. The SEI in the electrode surface layer consisted of both organic (ROCO_2_Li) and inorganic (LiF, Li_2_CO_3_) components. Specifically, in the F1s spectra, LiF and Li*_x_* PO*_y_* F*_z_* are the main products of the decomposition of the electrolyte, and LiF has intensive signals indicating the enrichment of this component in SEI. Notably, LiF has a high shear modulus, which can effectively resist the subsequent attack of electrolytes.^[^ ^30^ ^]^ Thus, it is a highly desirable composition in the SEI layer to help maintain a stable interface.^[^ ^31^ ^]^ Additionally, new peaks appearing at 290 eV in the C1s spectrum, as well as 531.8 and 531.3 eV in the O1s peak, correspond to the signal of ROCO_2_Li and Li_2_CO_3,_ respectively. The organic component ROCO_2_Li can enhance the flexibility of the SEI.^[^ ^32^ ^]^ While Li_2_CO_3_ can help form a continuous and smooth structure of the SEI which will promote the lithium‐ion diffusion ability.^[^ ^33^ ^]^ From Figure S15a–c and Table S2, Supporting Information, it is noticed that there is negligible change in the SEI composition from the 5th to 500th cycles, indicating the good stability of the interface in the Si@GCA13 anodes. All these results clearly demonstrate that, by performing a CA‐rich coating on the Si surface, this multifunctional GCA13 binder can help induce a compatible and stable SEI (after five cycles) to further promote the long cycling performance of the electrode.

**Figure 4:**
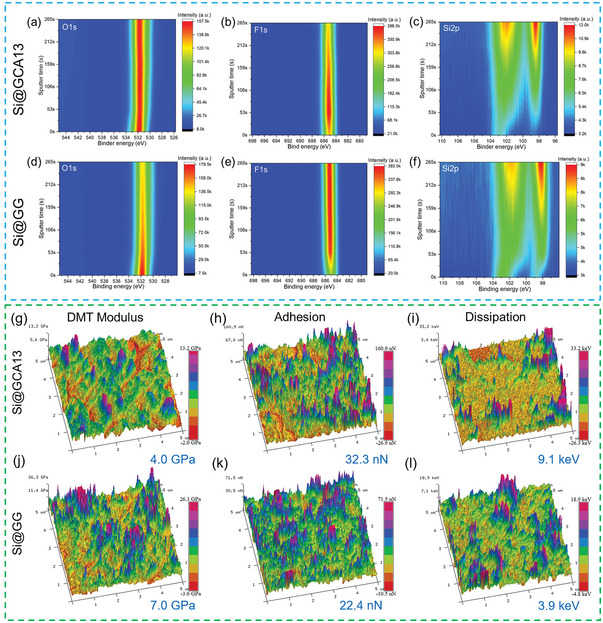
Contour maps of the O 1s, F 1s, and Si 2p intensity for a–c) Si@GCA13 and d–f) Si@GG under different sputtering times after 30 cycles. DMT modulus mapping of g) Si@GCA13 and j) Si@GG (j) electrodes after 30 cycles. Adhesion mapping of h) Si@GCA13 and k) Si@GG electrodes after 30 cycles. Dissipation mapping of i) Si@GCA13 and l) Si@GG electrodes after 30 cycles.

The composition gradient and structure of the SEI layer in the electrode were examined via XPS assisted with an Ar^+^ sputtering depth profiling (about 0.19 nm s^−1^), as shown in **Figure** **4**a–f; Figures S16 and S17, Supporting Information. With the increase of sputtering time, the spatial‐dimension element intensity contour map of the Si@GG electrode displays a LiF‐rich phase and intensity change of O1s. This uneven distribution of each component and excessively high F content is detrimental to the stability of SEI, and a relatively low Si2p signal indicates a thick SEI. In contrast, in Figure 4a–c, for the Si@GCA13 electrode, the intensity of O1s and appropriate LiF almost remains steady with the increase of sputtering depth, indicating that the Si@GCA13 electrode has a relatively stable and spatially uniform distribution of elements in SEI. Meanwhile, it reveals that the SEI of the Si@GCA13 electrode roughly shows a two‐layer structure. The thin outer layer shows intensive signals of organic groups, implying the containing of enriched organic components including ROCO_2_Li. These contents can enhance the elasticity and ductility of the SEI, which can be confirmed by the results in Figure 4g–l. While the inner layer of SEI is mainly composed of inorganic components, such as LiF and Li_2_CO_3_, LiF is an applicable component for SEI. On the one hand, due to its high interfacial energy with Li*_x_* Si and Li_4_SiO_4_ (main and side products of Si lithiation), the LiF‐rich SEI has low adhesion to the Si cores so that can alleviate the destruction of the SEI layer from Si/Li*_x_* Si huge volume change during cycling.^[^ ^31a^ ^]^ On the other hand, the wide band gap and insulating properties of LiF reduce the thickness of SEI.^[^ ^34^ ^]^ Meanwhile, the component of Li_2_CO_3_ can help induce a continuous and smooth structure in SEI.^[^ ^35^ ^]^ This presence of Li_2_CO_3_ may benefit the Li^+^ diffusion efficiency due to its low Li^+^ diffusion energy barriers resulting from the abundant Li vacancies.^[^ ^33^ ^]^ In addition, appropriate organic components can effectively improve the mechanical properties of SEI. Therefore, this compatible double‐layered SEI induced by the GCA13 binder is beneficial to the interface properties of the electrode both mechanically and electrochemically.

To clarify the mechanisms of the superior structural and interfacial stability, the mechanical properties of SEI were further investigated by AFM measurements using the Peak Force Quantitative nanomechanical (QNM) mode. A Derjaguin–Muller–Toporov (DMT) fitting model was employed to analyze the elastic modulus of the SEI (on the top surface of the electrode). As shown in Figure 4g,j, the DMT modulus of the Si@GCA13 electrode (4 GPa) is much smaller than that of the Si@GG electrode (7 GPa), revealing better flexibility and ductility of SEI in Si@GCA13. Meanwhile, in Figure 4h,k, the adhesion of the Si@GCA13 electrode (32.3 nN) to the probe tip is higher than that of the Si@GG electrode (22.4 nN). It suggests that, though the adhesive strengths of these two electrodes are comparable before cycling (Figure S8, Supporting Information), the cycled Si@GCA13 anode experienced an increase in adhesion property due to its unique SEI composition and the self‐tuned structure. Comparing the data in Figure 4i,l, the dissipation energy of the Si@GCA13 (9.1 keV) electrode is much higher than that of the Si@GG electrode (3.9 keV). A high energy dissipation implies a high tendency to occur plastic deformation in the electrode. Specifically, benefiting from the viscoelastic binder and the ductile SEI it induced, the Si@GCA13 anode has an excellent ability to adapt Si volume change through particle rearrangement or SEI deformation to release stress. Thus, the advanced elasticity, adhesion, and ductility of the SEI layer can further promote the mechanical properties of the Si@GCA13 electrode, and therefore, benefit the cycling performance.

**Figure 5:**
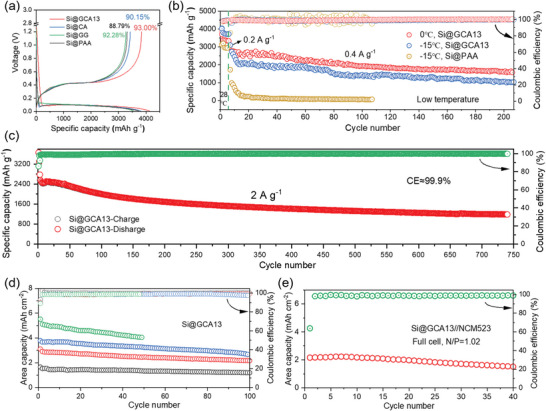
a) The initial coulombic efficiency and charge–discharge capacity–voltage curves of electrodes with different binders. b) Cycling performance of Si@GCA13 and Si@PAA electrodes at low temperatures (0 and −15 °C). c) Cycling stability of Si@GCA13 electrode at a current density of 2 A g^−1^. d) Cycling performance of Si@GCA13 electrode with different areal capacity. e) Cycling stability of Si@GCA13//NCM523 full cell at 0.1 C.

The electrochemical stability of the pristine GCA13 binder was analyzed by linear sweep voltammetry (LSV) and cyclic voltammetry (CV). From Figure S18, Supporting Information, no significant oxidation current was found before 3.9 V, indicating the electrochemical stability of the pristine GCA13 binder within 0.01–3.9 V. This can be confirmed by the CV curves in Figure S19, Supporting Information. Moreover, the CV curves of Si anodes prepared by the GCA13 binder were collected at 0.01–2 V in Figure S21, Supporting Information. In the first cathodic scan, a sharp peak appeared around 0.01 V is attributed to the lithiation of the crystalline Si.^[^ ^36^ ^]^ In the subsequent cathodic scans, the appearance of new reduction peaks at near 0.19 V are corresponding to the reversible lithiation of amorphous silicon to the Li*_x_* Si phase.^[^ ^37^ ^]^ The two oxidation peaks arose around 0.37 and 0.53 V is attributed to the delithiation of the Li‐Si phase.^[^ ^38^ ^]^ Similar peaks were also observed in the Si anodes prepared by GG and CA binder. Besides, the galvanostatic charge–discharge tests were employed on the Si anodes prepared with different binders to compare their electrochemical properties. During the initial cycle at the current density of 0.2 A g^−1^, all Si anodes show similar lithiation and delithiation plateaus at about 0.1 and 0.4 V (**Figure** **5**a), which agree with the CV curves. Concerning the initial coulombic efficiency (ICE), which is crucial for the practical application of silicon anodes, the Si@GCA13 electrode displays the highest ICE of 93%, indicating that the GCA13 binder has a great superiority in the stabilization of the interface via alleviating the side reactions.^[^ ^39^ ^]^

Figure 5b shows the long‐term cycling performance of the Si@GCA13 anodes at various low temperatures. It can be seen that the electrodes deliver a stable reversible capacity of 1578 mAh g^−1^ after 200 cycles and 1025 mAh g^−1^ after 200 cycles at 0 and −15 °C, respectively, which are outstanding among published data (Table S3, Supporting Information). Furthermore, the Si@GCA13 electrode also shows a high capacity of 2302 mAh g^−1^ after 55 cycles at 60 °C (Figure S22, Supporting Information). These results illuminate that Si anodes prepared with the GCA13 binder have a great potential to use in harsh environments, which is essential in real applications. This unique advantage can be attributed to the self‐tuning and self‐healing abilities of the electrodes, which derive from the viscoelasticity of the GCA13 binder. The cycling performance of silicon electrodes with different binders at room temperature is evaluated in Figure S23, Supporting Information. At the current density of 0.8 A g^−1^, the Si@GG and Si@PAA electrodes show steep decays during cycling due to their rigid polymer chains, which are unable to withstand the Si volume variation. The failure of the Si@CA electrode after 200 cycles is attributed to the lack of long‐range backbones in the binder network. In contrast, the Si@GCA13 electrode shows a high discharge capacity of 2258 mAh g^−1^ and capacity retention of 62% (300th/fifth). These excellent properties benefit from the synergistically long‐range and short‐range effects of the GCA13 binder, which construct a robust network to mechanically and electrochemically support the electrode.

A binder with high ionic conductivity can substantially enhance the cyclic performance of the Si anode, especially for the rate performance.^[^ ^38^ ^]^ Figures S24 and S25, Supporting Information, show the *D*_Li+_ of Si@GCA13 anode varies from ≈2.288 × 10^−10^–5.516 × 10^−9^ cm^−2^ s^−1^ during charge and discharge, which is larger than those of Si anodes using others binders. The fast Li^+^ diffusion efficiency of the Si@GCA13 anode indicates that based on the synergistic effect of the long‐range function (Li^+^ transport highway along the GG backbones) and the short‐range function (local interactions induced by CA molecules), this GCA13 binder can maintain an integrated network for ionic conduction. Regarding the rate performance in Figure S26, Supporting Information, the Si@GCA13 electrode show high specific capacities of 4015, 3682, 3412, 2976, and 2416 mAh g^−1^ at 0.2, 0.5, 1, 2, and 4 A g^−1^, respectively. When the current density changes to 0.2 A g^−1^, the specific capacity recovers to 3897 mAh g^−1^, confirming the outstanding structural stability and Li^+^ transport kinetic properties of this anode.

Additionally, the Si@GCA13 electrode also displays a stable long‐term cycling performance. It maintains a high capacity of 1184 mAh g^−1^ after 740 cycles at 2 A g^−1^ with a quick rise of CE (>98.6% after three cycles) (Figure 5c). The cycling stability of the silicon electrodes with high areal capacities was further investigated in Figure 5d. The Si@GCA13 electrode displays a high areal capacity of 4 mAh cm^−2^ after 50 cycles and exhibits excellent cycling performance for 100 cycles at both 3.6 and 2.9 mAh cm^−2^. Moreover, the full cell pairing with NCM523 achieves a high reversible areal capacity of about 1.5 mAh cm^−2^ after 40 cycles, demonstrating the good potential for practical application in LIBs (Figure 5e). These outstanding electrochemical performances of the electrodes verify the superiorities of the multi‐functional GCA13 binder. Based on the self‐tuning and self‐healing mechanisms, this viscoelastic binder can remarkably promote the overall properties of the Si anodes via structural and interfacial stabilization.

## 3 Conclusion

In summary, a multi‐functional binder (GCA13) was designed based on the synergistic strategy of the robust long‐range (derived from the GG backbones) and flexible short‐range (induced by the CA small molecules) effects for the Si anodes. Other than the conventional polymeric binder, by plasticization of small molecules, this viscoelastic binder gives rise to the electrode a remarkable self‐tuning and self‐healing ability via Si particle rearrangement. Therefore, the Si@GCA13 electrode can maintain an integrated and stable structure during cycling. Moreover, by performing a CA‐rich coating on the Si surface, this binder induces a compatible double‐layered SEI to construct an electrochemically stable interface with appropriate modulus and energy‐dissipating properties. Consequently, the Si@GCA13 electrode exhibits outstanding electrochemical performance in terms of long‐term cycling, full‐cell cycling, and under practical areal capacity. Particularly, the unique properties of the GCA13 binder ensure the service of the Si anodes in a very wide temperature range (from −15 to 60 °C). This work shows that an appropriate design of the binder can effectively promote the properties of the Si anodes from all‐around aspects. It is expected that this particle self‐tuning mechanism and the green fabrication approaches can contribute to the development of high energy density batteries with good safety and long life for practical applications.

## Conflict of Interest

The authors declare no conflict of interest.

## Supporting information

Supporting Information

## Data Availability

The data that support the findings of this study are available from the corresponding author upon reasonable request.
