# Supplementary material for: An Energy Dissipative Binder for Self‐Tuning Silicon Anodes in Lithium‐Ion Batteries
Source: Adv Sci (Weinh). 2022 Nov 17;10(2):2205443. doi: 10.1002/advs.202205443 (PMC9839849; doi:10.1002/advs.202205443)
Supplement: Supplementary file 1 — Supporting Information [file ADVS-10-2205443-s001.pdf]

ADVANCED SCIENCE
Open Access

Supporting Information

for *Adv. Sci.*, DOI 10.1002/advs.202205443

An Energy Dissipative Binder for Self-Tuning Silicon Anodes in Lithium-Ion Batteries

Yihong Tong, Siyu Jin, Hongyuan Xu, Jiawei Li, Zhao Kong, Hong Jin and Hui Xu**

An Energy Dissipative Binder for Self-tuning Silicon Anodes in Lithium-ion Batteries

Yihong Tong, Siyu Jin, Hongyuan Xu, JiaWei Li, Zhao Kong, Hong Jin and Hui Xu**

Y. H. Tong, H. Y. Xu, J. W. Li, Z. Kong, H. Jin, H. Xu
Suzhou Academy,
Xi'an Jiaotong University
Suzhou 215123, China
E-mail: xhxuhui@xjtu.edu.cn, jhjinhong@xjtu.edu.cn
Y. H. Tong, H. Y. Xu, J. W. Li, Z. Kong
Nano Science and Technology Institute
University of Science and Technology of China
Suzhou 215123, China
Y. H. Tong, H. Y. Xu, J. W. Li, Z. Kong
Suzhou Institute for Advanced Research
University of Science and Technology of China
Suzhou 215123, China
S. Y. Jin
Sustainable Energy Laboratory
Faculty of Materials Science and Chemistry
China University of Geosciences
Wuhan 430074, China

Experimental Section

Materials. Guar gum (GG) and polyacrylic acid (PAA) were purchased from Aladdin. Citric acid monohydrate (CA.H_2O) was purchased from Sinopharm. SiNPs (about 100 nm) was purchased from Zhejiang Zhongning Polysilicon Co., Ltd., and the XRD of Si particles showed that all peaks were consistent with standard crystallographic data (Figure S1).

Preparation of Si anode. Firstly, guar gum and citric acid monohydrate were added in appropriate amount of deionized water, and the mixture was magnetically stirred at 60 °C for 30 min. After that, active materials and conductive materials was added to the above solution to prepare a uniform slurry. Secondly, the Si anodes are prepared by casting the slurries including active materials (80*wt*%, Si powder), conductive materials (super P and SWCNTs are 4*wt*% and 1*wt*%, respectively) and binder (15*wt*%) on the copper foil. Thirdly, the as-prepared electrodes were placed at room temperature for a period of time, and then placed in an ultra-low temperature refrigerator (about -75 °C, Thermo Fisher 905-ULTS) for 6 h. Finally, freeze-dry (LABCONCO, 2.5L Freeze Dry) the above membrane for about 20 h to obtain the final electrode. The control electrode was fabricated with reference to this process.

Materials characterization. SEM and TEM images were obtained by HITACHI SU8010 and Talos F200x, respectively. FTIR (Thermo Scientific Nicolet iS20) was used to characterize the chemical composition of samples, and XRD (Burker D8 Advance) were used to analyze the raw materials. The viscoelasticity of GCA13 binder was verified using Mars40 (Thermo Fisher) instrument, and the wettability was analyzed by testing the contact angle (JY-82B Kruss DSA). XPS (Thermo Scientific K-Alpha, Al Kα) was used to analyze the composition, in addition, the composition at different depths of the SEI was explored by etching XPS. The peak force mapping AFM (Dimension Icon, Bruker) experiments were performed to measure the surface topography, roughness, Young’s modulus, adhesion and dissipation. The Young’s modulus, adhesion and

dissipation of Si@GCA13 and Si@GG electrode after 30 cycles were derived from the gained force curves based on the DMT model. Optical microscopy (Olympus BX51M) was used to observe the changes in cracks before and after electrode cycling. The adhesion and tensile tests were carried out by the Electronic Universal Testing Machine at a stretching speed of 50 mm min^{-1}.

Electrochemical Characterization. The 2032 coin-type half-cells were assembled in an argon filled glovebox with oxygen and water content below 1 ppm. The above-mentioned dried electrode was pressed into a disc with a diameter of 12 mm with a mass of about 0.6~1.4 mg cm^{-2} of active material as the working electrode, and metal lithium foil was used as the counter electrode. A Celgard 2400 apparatus was used as the separator, and 1 M $LiPF_6$ in EC: DEC=1: 1, add 10%FEC and 1%VC as the electrolyte was used. The as-assembled cells were aged for 24 h before the galvanostatic charge-discharge cycles (voltage 0.01~1.2 V) were tested on a Neware CT-3008-S4 station. Linear sweep voltammetry (LSV) and Cyclic voltammetry (CV) were also conducted on a CHI660E electrochemical workstation at a scanning rate of 0.1 mV s^{-1}. The galvanostatic intermittent titration technique (GITT) were performed on a Neware CT-3008-S4 station at room temperature under the voltage range of 0.01-1.2 V, and relaxation time of 30 min and current density of 0.8 A g^{-1}. Particularly, the long-term cyclic performance of Si@GCA13 electrodes at low temperature were tested in a refrigerator due to the lack of experimental equipment, the data at 0 °C and -15 °C in the text were obtained at -7~0 °C and -17~-14 °C, respectively. The batteries were frozen in the refrigerator for more than 12 hours before testing.

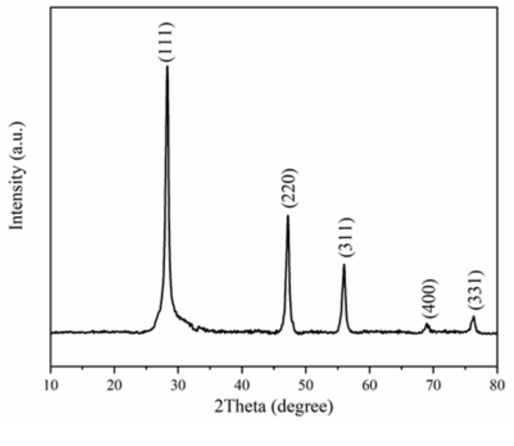

Figure S1. The XRD pattern of the SiNPs.

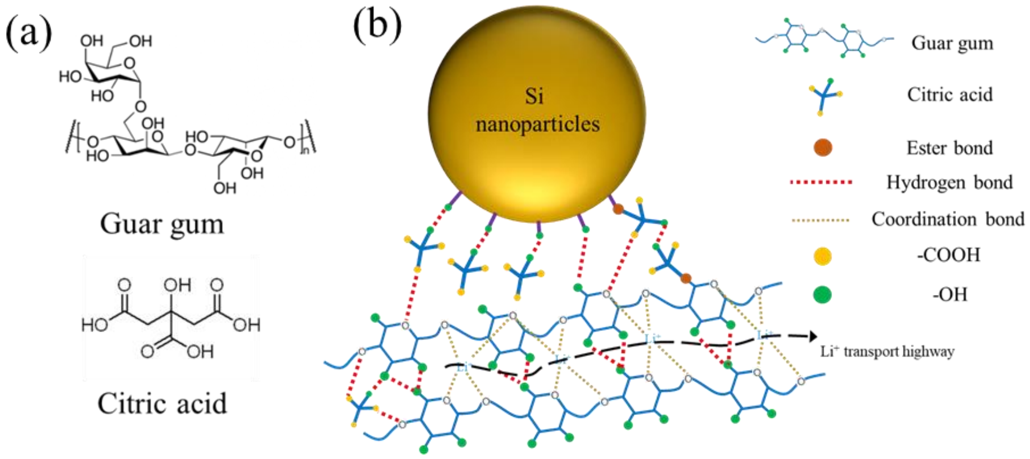

Figure S2. (a) Chemical structures of guar gum and citric acid. Schematic diagram of chemical interaction between binder and silicon (b)

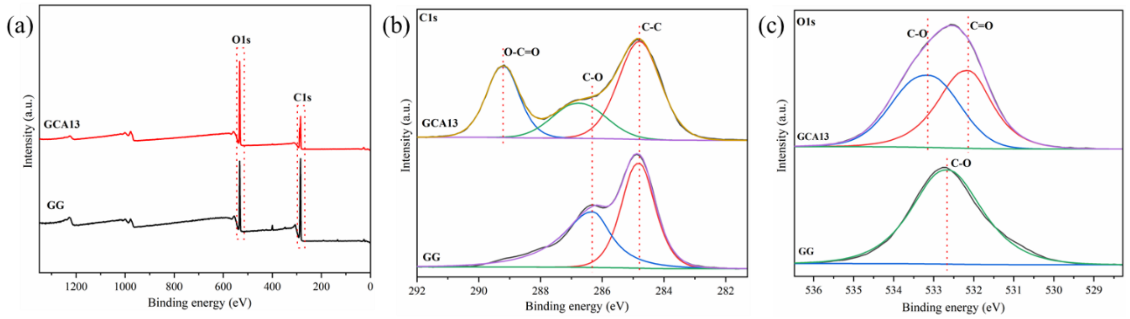

Figure S3. The XPS spectra of GCA13 and GG, (a) full spectrum, high-resolution spectrum of C1s (b), O1s (c).

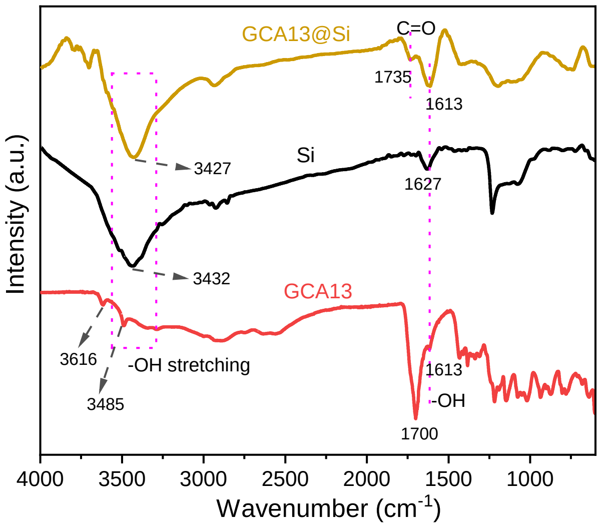

Figure S4. FTIR spectra of GCA13, Si and GCA13@Si.

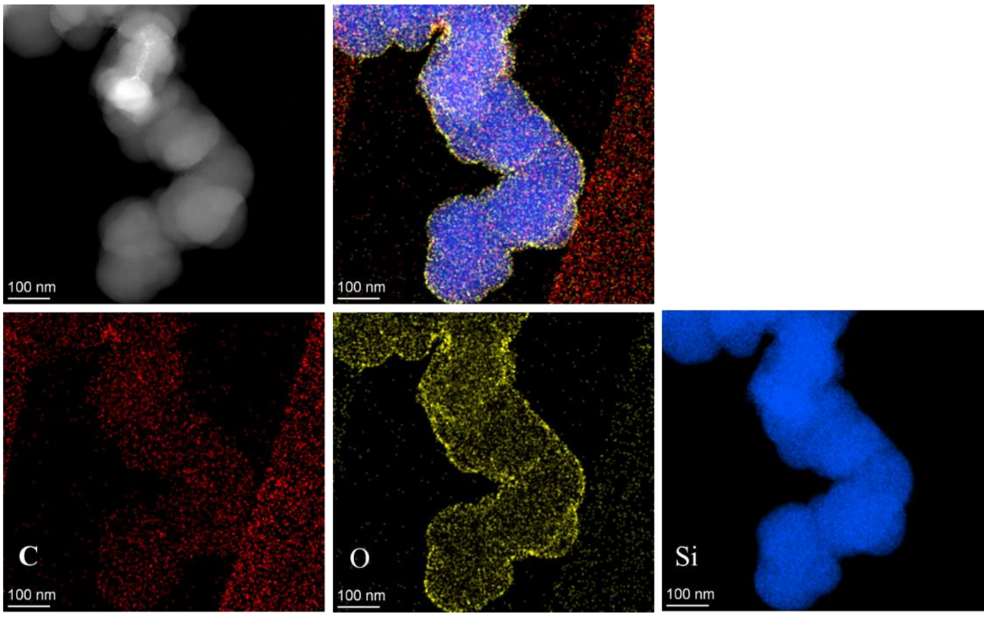

Figure S5. TEM elemental mapping images of C, O and Si from Si@GCA13 particles.

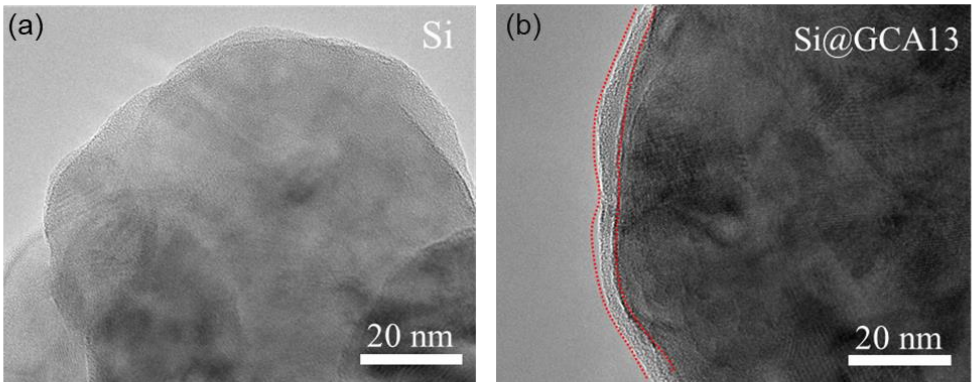

Figure S6. TEM images of pristine (a) and Si@GCA13 (b).

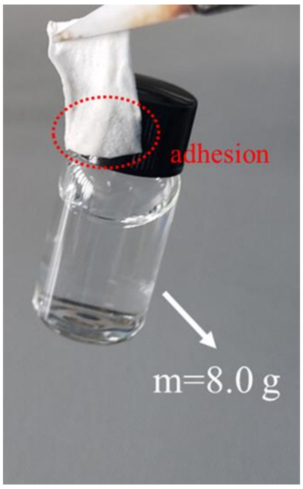

Figure S7. Digital photographs of adhesion test of GCA13 polymer film.

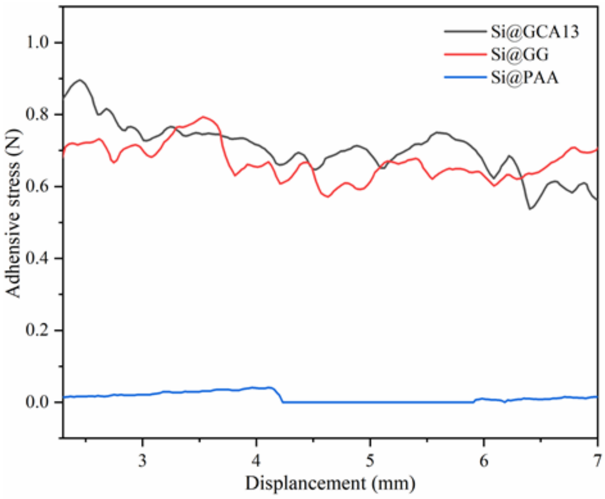

Figure S8. The 180° peel test curve of Si@GCA13, Si@GG and Si@PAA electrodes.

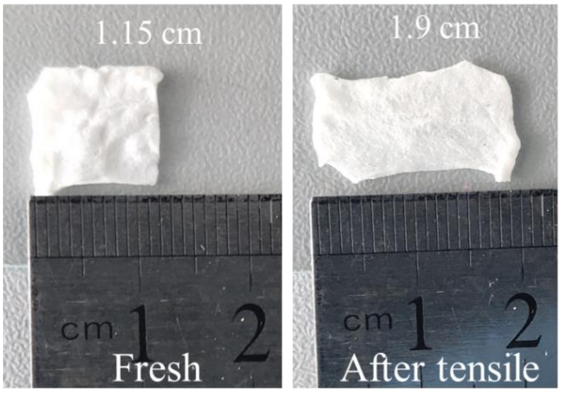

Figure S9. Digital photographs of stretch of GCA13 polymer film.

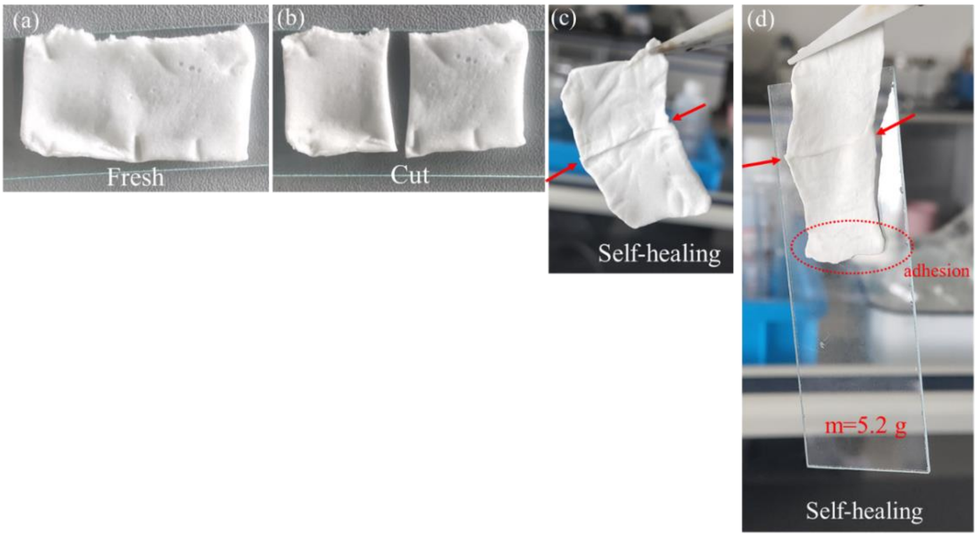

Figure S10. Digital photographs of self-healing performance evaluation of GCA13 polymer film (a-d).

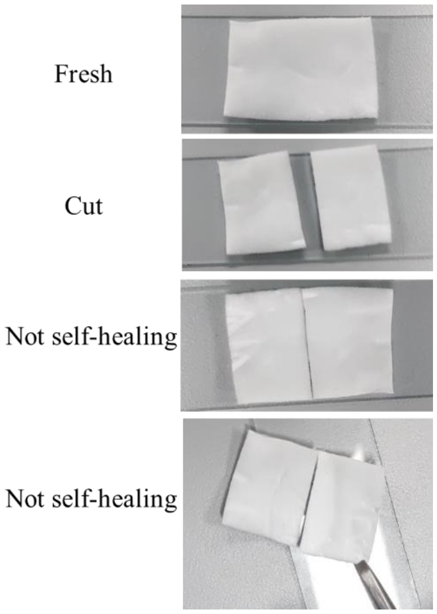

Figure S11. Digital photographs of GG polymer film.

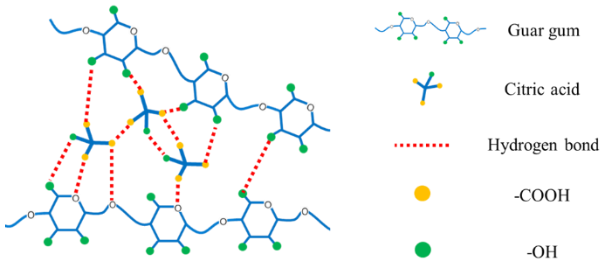

Figure S12. Schematic diagram of multiple hydrogen bonds in GCA13 polymer film.

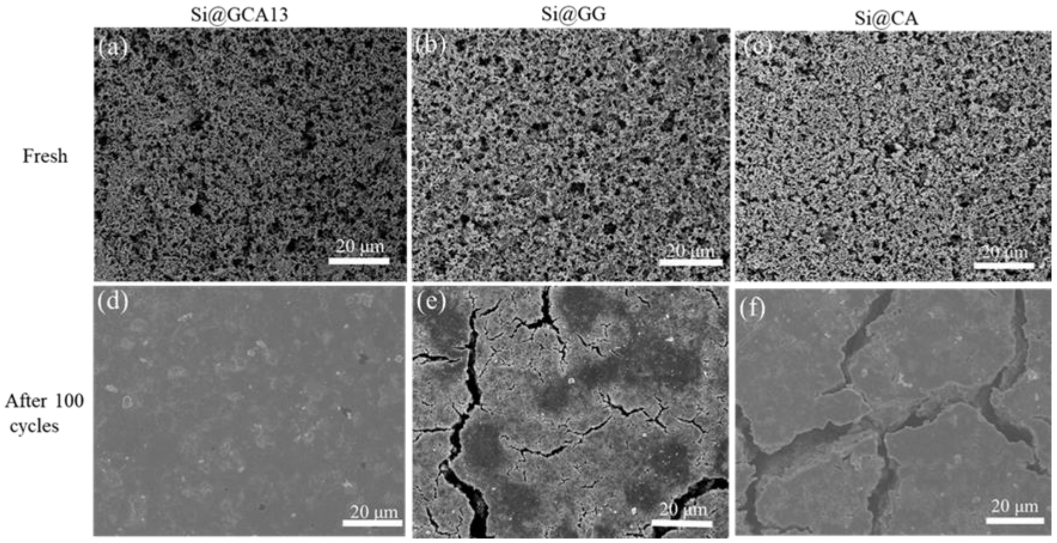

Figure S13. Top-viewed SEM images of fresh GCA13 (a), GG (b) and CA (c) electrode. Top-viewed SEM images of GCA13 (d), GG (e) and CA (f) electrode after 100cycles. After 100 cycles, the Si@GCA13 electrode showed no cracks on the surface, while the Si@GG and Si@CA electrodes developed large cracks and the structure collapsed completely.

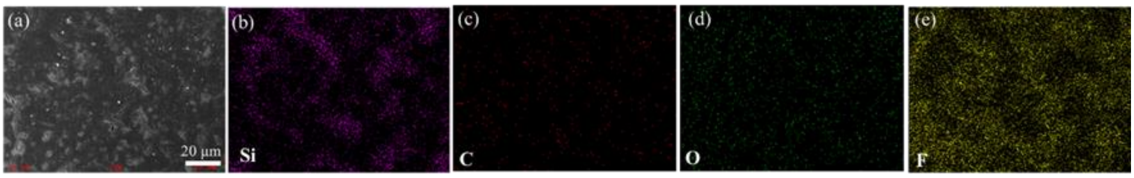

Figure S14. Element mapping of Si@GCA13 electrode after 100 cycles.

Table S1. Comparison with previous literature

Binder	Si: binder	Cycle number	Thickness change rate.	Ref.
PAA-BFPU	70: 15	50	149%	[1]
PAA			293%	
PVDF			303%	
DNB	6: 2	30	170%	[2]
Pectin			280%	
PAA			275%	
N-P-LiPN	80: 10	20	150%	[3]
P-LiPAA			186%	
P-LiNF			220%	
GCA13	80: 15	100	158%	This work
GG			226%	
GCA13		500	184%	

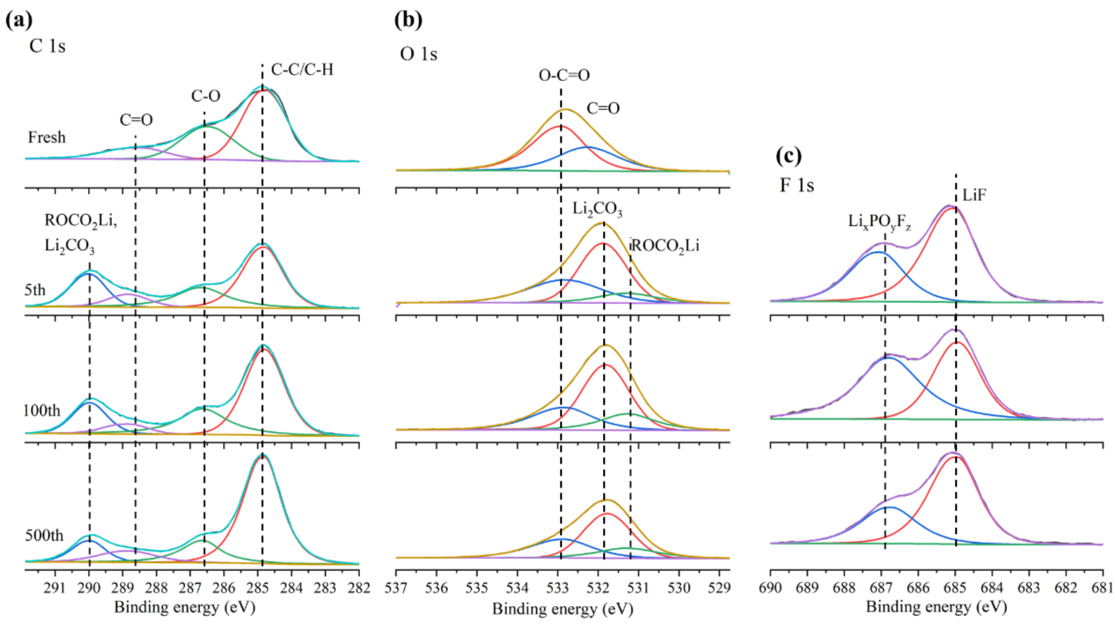

Figure S15. XPS spectra of Si@GCA13 electrode at different cycles. (a) the C1s spectra, (b) the O1s spectra and (c) the F1s spectra.

Table S2. XPS spectra fit peak values.

Cycles	Peaks (eV)		
	C1s	O1s	F1s
Fresh	284.8, 286.5, 288.5	532.3, 532.9	
5th	284.8, 286.7, 288.8, 290.0	531.3, 531.8, 532.8	685.0, 687.0
100th	284.8, 286.6, 288.8, 290.0	531.3, 531.8, 532.8	685.0, 686.8
500th	284.8, 286.6, 288.8, 290.0	531.3, 531.8, 532.9	685.0, 686.7

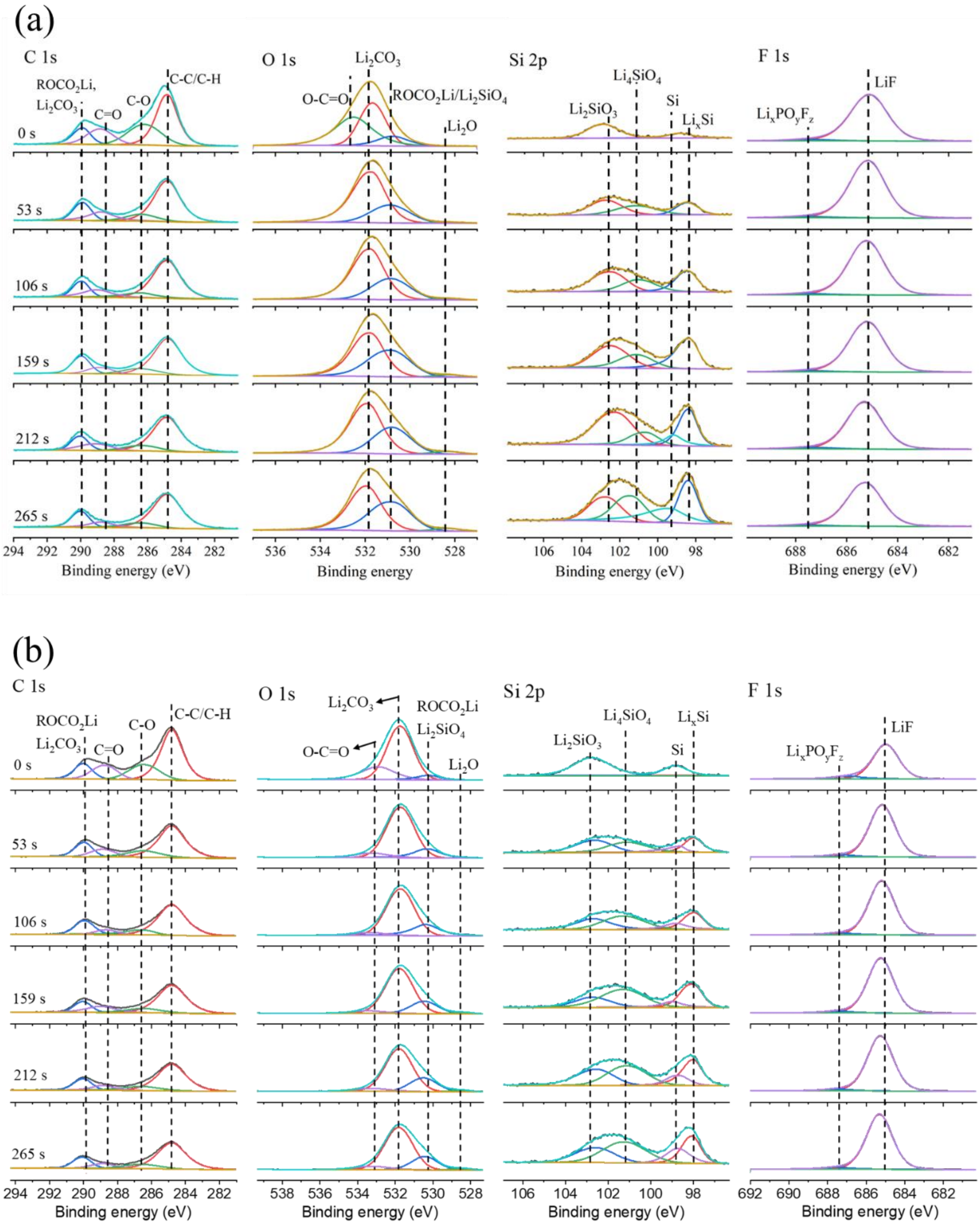

Figure S16. XPS characterization of the SEI formed on Si@GCA13 (a) and Si@GG (b) electrodes after 30 cycles. The C1s, O1s, Si2p and F1s spectra are showed in columns, which show the corresponding depth profiling results.

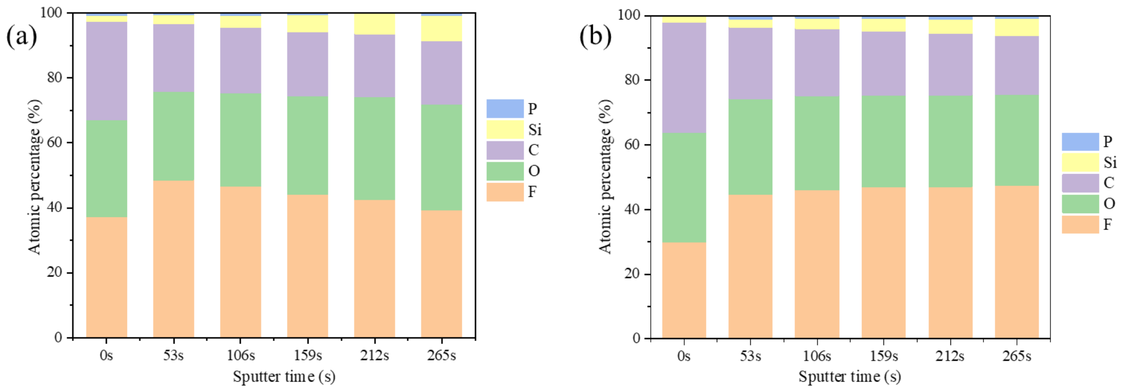

Figure S17. Atomic compositions of Si@GCA13 (a) and Si@GG (b) anodes after 30 cycles (after 0, 53s, 106s, 159s, 212s and 265s of Ar^+ sputtering).

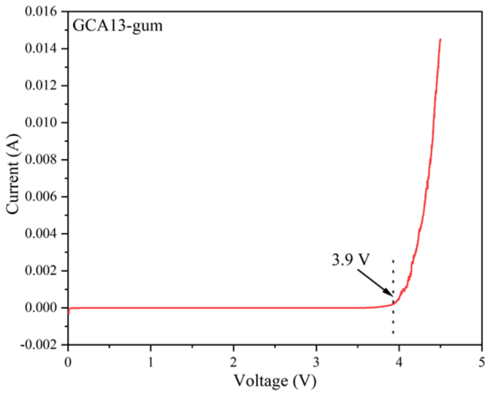

Figure S18. Linear sweep voltammetry (LSV) curve of the GCA13 binder at 0.1 mV s^{-1} (0.01~5.0 V).

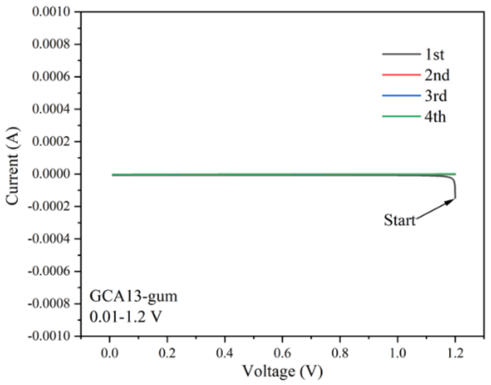

Figure S19. CV curves of GCA13 gum at 0.1 mV/s (first four cycles, 0.01~1.2 V). The absence of cathodic or anodic peaks confirmed the electrochemical stability of GCA13 binder at 0.01~1.2 V. Meanwhile, the current at the beginning is because the scan start voltage (1.2 V) is not the same as the open circuit voltage of half-cell (2.7 V).

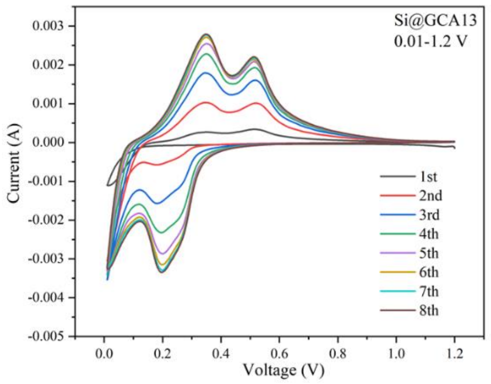

Figure S20. CV curves of Si@GCA13 at 0.1 mV s^{-1} (first eight cycles, 0.01~1.2 V).

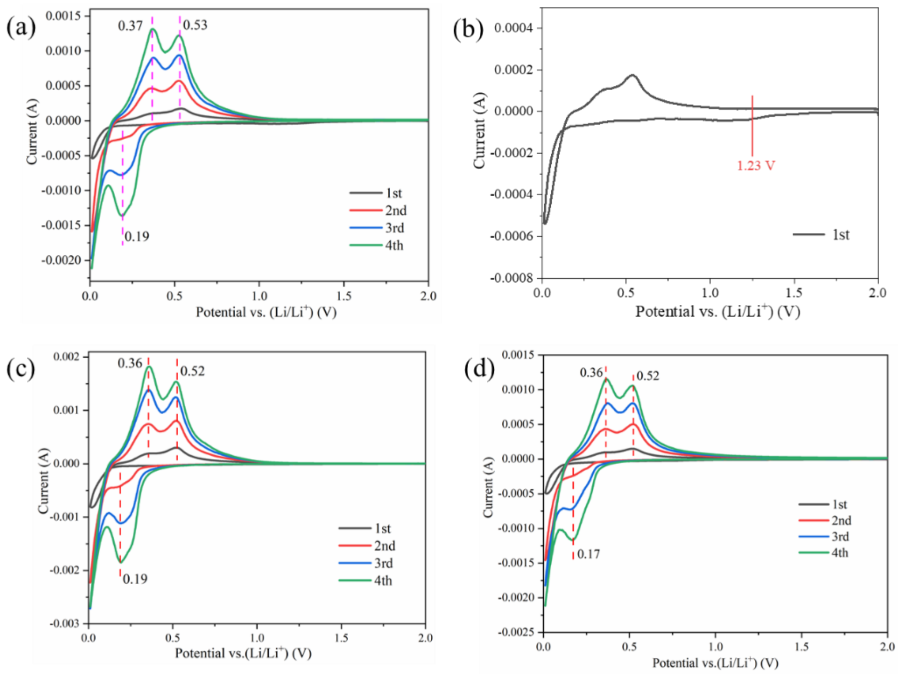

Figure S21 CV curves of Si@GCA13 (a, b), Si@GG (c) and Si@CA (d) electrode at 0.1 mV s^{-1} (0.01~2.0V).

Table S3. Comparison with previous literature.

Cathode‖anode	electrolyte	Temperature (°C)	Discharge capacity (mAh g^{-1})	Notes	Ref.
Si nanoflakes‖Li	1 M $LiPF_6$, EC/DEC (1 : 1 v/v)	-5	1679	After 50 cycles	[4]
Si nanopillars‖Li	1 M $LiPF_6$, FEC/DMC (1 : 4 w/w)	-10	600	Capacity-limited to 600 mAh g^{-1} charge	[5]
		-20	600		
		-30	600		
Si/Gr‖Li	1 M $LiPF_6$, EC/DEC/DMC/EMC (1 : 1 : 1 : 3 v/v)	-10	277	10% nano-Si, polymer/ ceramic composite binder	[6]
		-20	222		
		-30	160		
		-40	84		
Si nanoparticles‖Li	1 M $LiPF_6$, EC/DEC (1: 1), add 10%FEC and 1%VC	0	1578	After 200 cycles	This work
		-15	1025	After 200 cycles	

(Note: there are few studies on silicon anodes at low temperature, and they mainly focus on electrolytes.)

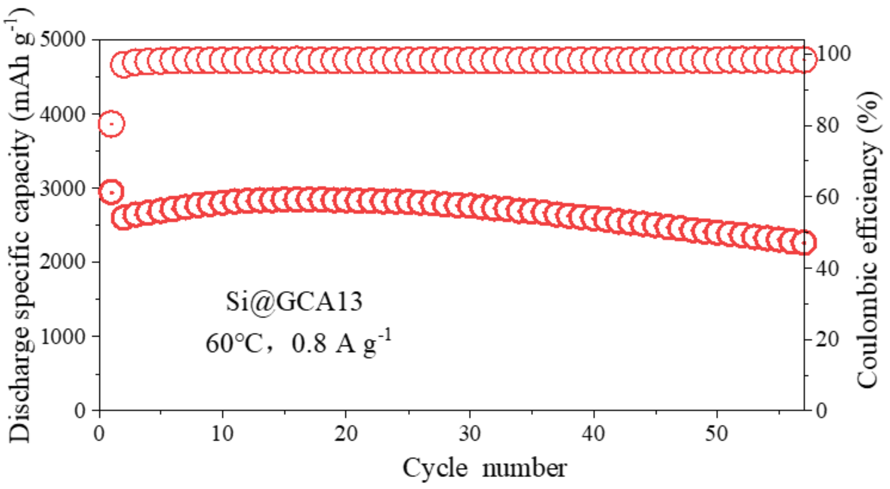

Figure S22. Cycle performance of Si@GCA13 electrode at 60 °C (0.8 A g^{-1}).

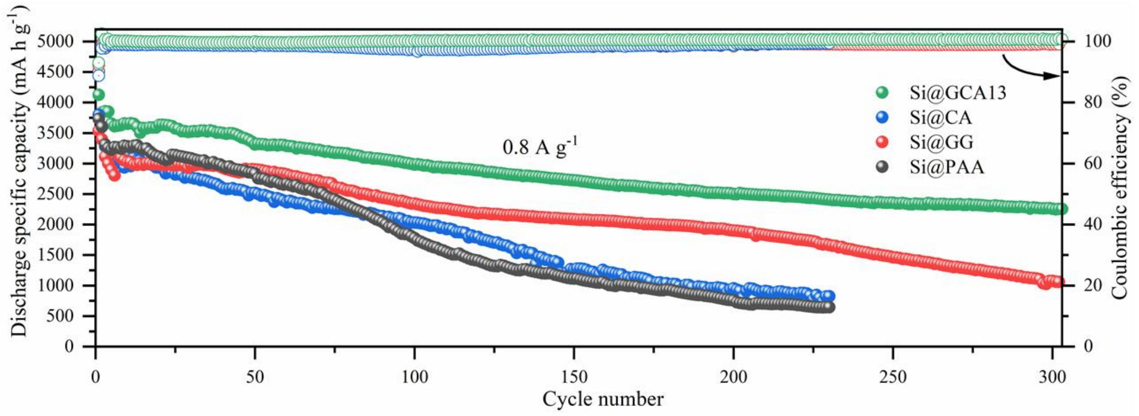

Figure S23. Long-term cyclic performance of Si electrode with different binders at 0.8 A g^{-1}.

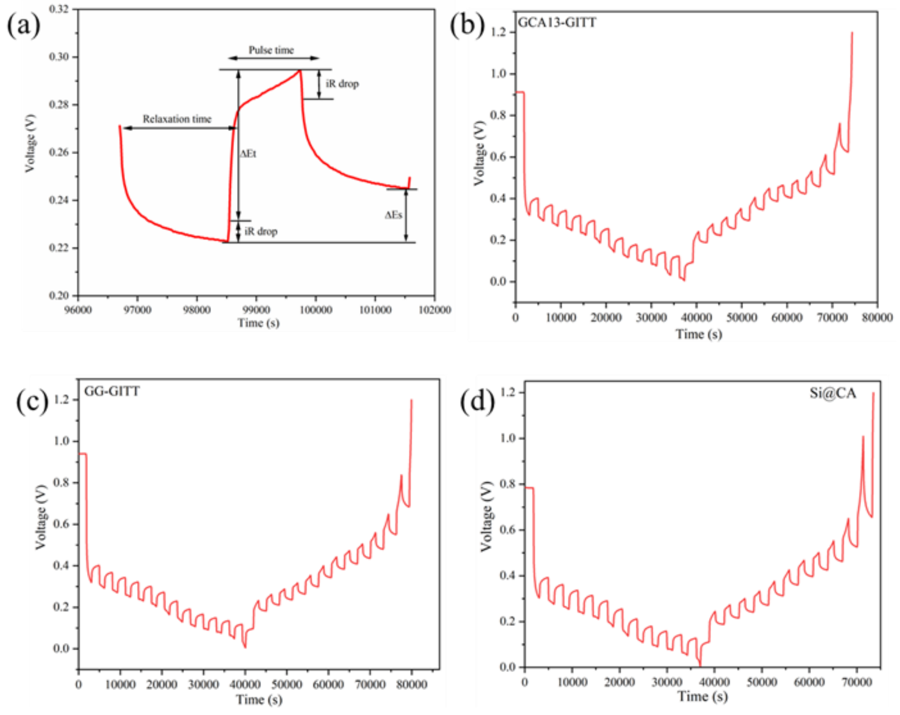

Figure S24. The GITT profiles of Si anode with different binder at 0.8 A/g. (a) Schematic diagram, (b) Si@GCA13 anode, (c) Si@GG anode, (d) Si@CA anode. All batteries are activated for 6 cycles before testing.

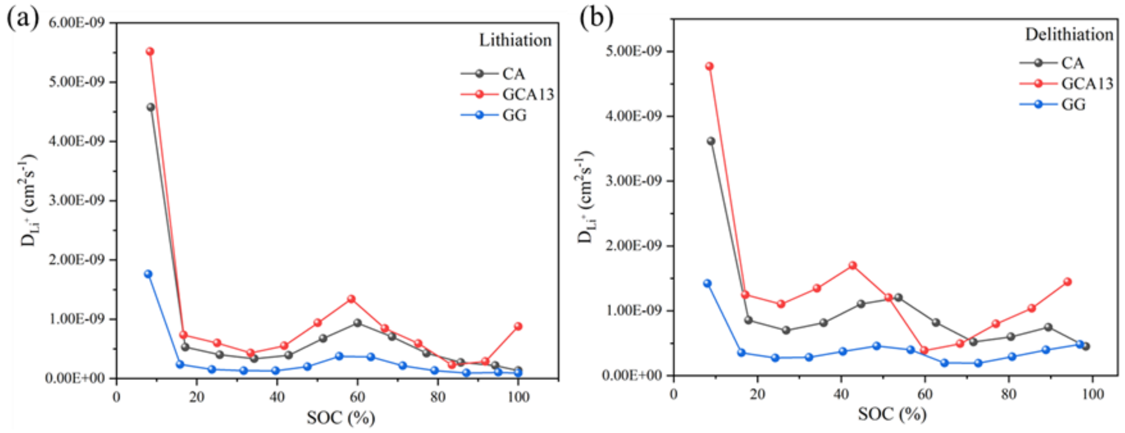

Figure S25. The D_{Li+} of Si electrode with different binder at 0.8 A/g (corresponds to the above GITT curve, Figure S20). The D_{Li+} of Si@GCA13 electrode varies from 2.288×10^{-10}~5.516×10^{-9} cm^{-2} s^{-1}, Si@GG electrode varies from 9.131×10^{-11}~1.761×10^{-9} cm^{-2} s^{-1}, Si@CA electrode varies from 1.344×10^{-10} ~4.575×10^{-9} cm^{-2} s^{-1}. The D_{Li+} of Si@GCA13 electrode is the highest here.

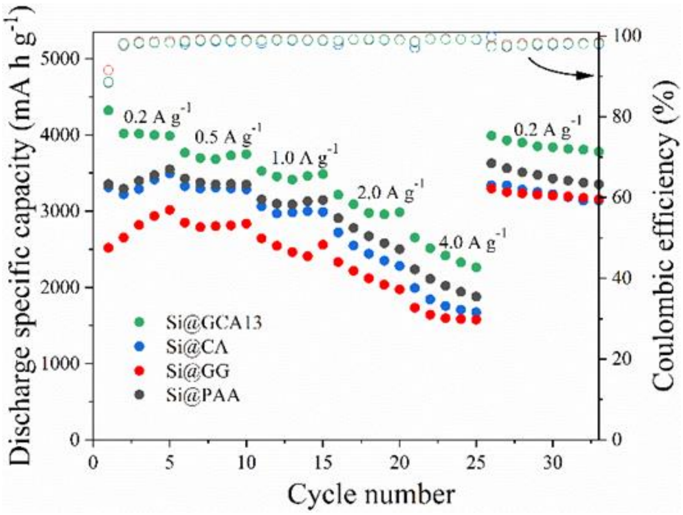

Figure S26. Rate performance of Si electrode with different binders.

[1] X. Jiao, J. Yin, X. Xu, J. Wang, Y. Liu, S. Xiong, Q. Zhang, J. Song, *Advanced Functional Materials* **2021**, 31.

[2] M. Jiang, P. Mu, H. Zhang, T. Dong, B. Tang, H. Qiu, Z. Chen, G. Cui, *Nano-Micro Letters* **2022**, 14, 87.

[3] Z. Li, Y. Zhang, T. Liu, X. Gao, S. Li, M. Ling, C. Liang, J. Zheng, Z. Lin, *Advanced*

Energy Materials **2020**, 10, 1903110.

[4] M. Haruta, T. Okubo, Y. Masuo, S. Yoshida, A. Tomita, T. Takenaka, T. Doi, M. Inaba, *Electrochimica Acta* **2017**, 224, 186.

[5] E. Markevich, G. Salitra, D. Aurbach, *Journal of The Electrochemical Society* **2016**, 163, A2407.

[6] D. Hubble, D. E. Brown, Y. Zhao, C. Fang, J. Lau, B. D. McCloskey, G. Liu, *Energy & Environmental Science* **2022**, 15, 550.
